# Epimetheus - a multi-profile normalizer for epigenomic sequencing data

**DOI:** 10.1186/s12859-017-1655-3

**Published:** 2017-05-12

**Authors:** Mohamed-Ashick M. Saleem, Marco-Antonio Mendoza-Parra, Pierre-Etienne Cholley, Matthias Blum, Hinrich Gronemeyer

**Affiliations:** 1 Equipe Labellisée Ligue Contre le Cancer, Department of Functional Genomics and Cancer, Institut de Génétique et de Biologie Moléculaire et Cellulaire, Illkirch, France; 2 Centre National de la Recherche Scientifique UMR 7104, Illkirch, France; 3 Institut National de la Santé et de la Recherche Médicale, U964, Illkirch, France; 4 Université de Strasbourg, Illkirch, France

**Keywords:** ChIP-seq, Epigenome, Quantile-based data normalization, Multi-sample analysis

## Abstract

**Background:**

Exponentially increasing numbers of NGS-based epigenomic datasets in public repositories like GEO constitute an enormous source of information that is invaluable for integrative and comparative studies of gene regulatory mechanisms. One of today’s challenges for such studies is to identify functionally informative local and global patterns of chromatin states in order to describe the regulatory impact of the epigenome in normal cell physiology and in case of pathological aberrations. Critically, the most preferred Chromatin ImmunoPrecipitation-Sequencing (ChIP-Seq) is inherently prone to significant variability between assays, which poses significant challenge on comparative studies. One challenge concerns data normalization to adjust sequencing depth variation.

**Results:**

Currently existing tools either apply linear scaling corrections and/or are restricted to specific genomic regions, which can be prone to biases. To overcome these restrictions without any external biases, we developed Epimetheus, a genome-wide quantile-based multi-profile normalization tool for histone modification data and related datasets.

**Conclusions:**

Epimetheus has been successfully used to normalize epigenomics data in previous studies on X inactivation in breast cancer and in integrative studies of neuronal cell fate acquisition and tumorigenic transformation; Epimetheus is freely available to the scientific community.

**Electronic supplementary material:**

The online version of this article (doi:10.1186/s12859-017-1655-3) contains supplementary material, which is available to authorized users.

## Background

Epigenetics is a complex and multi-layered process with potentially profound implications in cell fate decisions, including phenomena such as differentiation or tumorigenesis. With the advancements and cost-reduction in high-throughput sequencing, next generation sequencing (NGS) has become inevitable for epigenome research. Studying the epigenome and its dynamics involves sequencing to associate histone and DNA modifications to the genome of a particular cell with the aim of characterising the state of chromatin – the cumulated histone and DNA modifications in different genomic regions, over time during (patho)physiological processes (cell fate changes) and between different samples.

However, its assessment via chromatin immunoprecipitation is inherently prone to significant variability, posing different bioinformatic challenges for comparative studies - a general caveat in Big Data integrative analysis. Multiple factors like antibody efficacy, sequencing library accuracy and depth have a direct impact on data quality and thus on any downstream analysis. Therefore, it is imperative to evaluate the quality of data prior to comparative studies (see for example, www.ngs-qc.org) [1]. However, even high quality datasets generally exhibit significant technology/user-derived signal amplitude differences, which require normalization prior to comparative analysis.

While significant computational efforts have been made in the past for single ChIP-seq data analysis, sophisticated computational and experimental methods to correct technical variability among multi-sample ChIP-seq analyses is acquiring importance recently. Initially, a simple linear normalization approach was widely used, where the counts were represented relative to the total number of reads to correct the throughput differences. However, such linear normalization, as used by RNA-seq-based tools like DESeq [2] and EdgeR [3] does not address the inherent differences in signal-to-noise ratios among samples. As there can be a significant disproportionate fraction of reads falling in background regions, the normalization of the total number of reads can lead to biased results for certain regions and for the entire global profile. This proved linear normalization to be unsuitable for ChIP-seq. Further, Taslim et al.*,* proposed a two-step non-linear approach, based on a locally weighted regression (LOESS) method to correct such differences among ChIP-seq data [4]. LOESS’s restriction to pairwise normalization led us to develop Polyphemus [5], a multi-profile normalization approach for RNA polymerase II (RNA PolII) datasets based on quantile correction, a method widely used in microarray studies [6]. Since then, other quantile based normalization tools have been developed, including ChIPnorm [7] or Epigenomix, [8], both of which focus on the identification of differentially enriched regions or genes.

All the above-mentioned tools suffer from a number of important limitations, namely (i) their annotation dependency, (ii) their restriction to specific regions, (iii) less user-friendliness, especially for non-bioinformaticians, and (iv) their inability to produce output files that are compatible with downstream analyses. Moreover, the existing approaches are mostly intended for a particular analysis, thus their normalization outputs are not readily exportable to other tools for multi-dimensional sample analysis and require programming skills. To overcome all these restrictions, we developed Epimetheus, a quantile-based multi-profile normalization tool. The genome-wide normalization procedure applied by Epimetheus enables optimal processing of datasets from different enrichment patterns, including broad/sharp histone modification or PolII-seq profiles, chromatin accessibility profiles generated by FAIRE-seq [9] and ATAC-seq [10], DNase-seq [11] and DNA methylome profiles generated by MeDIP-seq or related approaches [12, 13]. Furthermore, users have the possibility to exclude specific genomic regions like, for example, repetitive elements or any other genomic locations for which artefactual enrichments might be expected.

## Methods

The basic assumption underlying quantile normalization is the presence of a common read-count distribution in the compared datasets. In cases where the compared enrichment events comprise factors that are implicated in house-keeping events, it is reasonable to assume that the distribution of the read counts for a given target will be similar across cell types [7].

As for gene expression analysis (RNA-seq and microarrays) or RNA polymerase II enrichment (Polyphemus [5]), where quantile has been widely used, histone modifications are expected to occur at both house-keeping and cell/tissue-specifically regulated genes. With this assumption, we apply genome-wide quantile normalization on multiple samples for each chromatin modification. Subsequently Z-score scaling is used, such that each dataset is represented relative to its mean of distribution, which renders different target histone data comparable. The Epimetheus pipeline involves four main steps: (i) processing of the raw alignment data, (ii) generation of read count intensity (RCI) matrices, (iii) computation of two subsequent levels of normalization (quantile and Z-score) and (iv) generation of outputs and plots (schematically depicted in Additional file 1: Figure S1).

### Processing of data

As quantile normalization is an absolute read count-based approach, any region-specific or technical bias will over/under-represent the read counts and lead to inaccurate downstream analyses. Clonal reads (i.e., PCR duplicates) constitute one such technical bias. Unfortunately, some level of clonal read contamination is unavoidable in sequencing datasets involving PCR. Epimetheus will remove such clonal reads from the raw alignment data, unless otherwise specified by the user. There are a few alignment and platform-specific biases that should be addressed prior to analysis as these are specific to each data and pipeline. Particularly recommended is to remove reads with more than one perfect alignment and those aligned to repeat and centromere regions. Also, a user can opt to exclude tricky regions from analysis using the respective option available in Epimetheus. Reads are elongated to a specified length to represent the average fragment length (150-300 bp), as typically only the first 50–100 base pairs are sequenced in ChIP-seq.

### Read count intensities

For quantile normalization, an approach similar to that of Xu et al. [14] and Mendoza-Parra et al., [5] is followed, where the reference genome ‘G’ (or custom regions for target-specific normalization) is divided into small non-overlapping sequential bins and the RCI for each bin is calculated. The size (‘S’) of the bin can range from 100 ≥ S ≤ 500 bp depending on the enrichment pattern (sharp/broad) of the histone mark. This bin size range is optimal to preserve the shape of enrichment patterns but users can nevertheless choose larger bin sizes, if required.

If X is a target histone mark and X_a_ & X_b_ are two samples of same target, then genomic bins for X_a_ will be x_a1_, x_a2_, x_a3_…x_an_ and for X_b_ they will be x_b1_, x_b2_, x_b3_…x_bn_, where ‘n’ depends on the sizes of ‘S’ and ‘G’ (G/S). Reads in each bin are counted to calculate the reads per bin (RpB) distribution for each sample, thus generating the two libraries X_a_ = {x_ai_ | 1 ≥ i ≤ n} and X_b_ = {x_bi_ | 1 ≥ i ≤ n}. Similarly, Y_a_ and Y_b_ will specify two different libraries for another target profile.

### Normalization

Using RCI calculation results, a B × N matrix is built, where B is the total number of bins (for a given ‘G’ and ‘S’) and N is the number of samples. In case of multiple histone marks, B × N1, B × N2, etc., will be similarly generated. The differences in coverage among samples are adjusted to same level by (i) sorting each sample’s RpB in ascending order individually, (ii) ranking the values for each sample individually, (iii) calculating the average of the corresponding rank values and (iv) assigning normalized values back to the original positions. This results in a normalized matrix norm(B × N), where each sample has normalized-RpB (nRpB). Subsequently, Z-score scaling is applied to the normalized matrix to generate znorm(B × N), which is calculated from the distance of each nRpB to a mean value of total nRpB in the sample, divided by the standard deviation.

Note that quantile-based normalization cannot be applied to profiles of different histone marks, as the signal distribution and amplitude can be highly dissimilar, which is incompatible with the initial assumption. Similarly, users should not apply quantile normalization in cases where major differences are expected between the datasets. For example, inhibitors of epigenetic enzymes are likely to exert global effects histone mark deposition [15]; quantile normalization of such systematically divergent profiles will very likely inappropriately alter the enrichment pattern. In principle, quantile normalization cannot not be applied to profiles with highly divergent patterns. An example is the estrogen receptor, a ligand-inducible transcription factor [16] which does not significantly bind chromatin in the absence of cognate estrogens; any normalization would artificially alter the compared profiles. Finally, normalization requires prior quality assessment for the same reasoning; a universal quality assessment tool and a large database (www.ngs-qc.org) can be consulted for guidance [1].

### Output

In contrast to previously described methods, Epimetheus produces normalized BED files by adding/removing reads with respect to normalized per-bin RCIs using raw alignment BED files as reference. Increasing counts is done by adding new reads aligned randomly to a new position within the concerned bin; existing reads are randomly removed to decrease read counts. As the BED format is the preferred input format in most of the ChIP-seq tools, Epimetheus enables the direct use of normalized data for downstream analysis.

Along with normalized BED output, Epimetheus produces three additional types of outputs: (a) visualization files, (b) plots and (c) normalized BED files. Visualization files are text files (in bedgraph format) generated for raw and normalized RCI, which can be used for other downstream analyses as well. To assess the difference among samples and the effect of normalization, MA transformation plots [17] are generated to compare samples pairwise before and after normalization. The tool is also capable of generating read counts matrix for targeted regions (promoter/gene-body/custom) and average RCI plots for the same.

## Results

Before initiating normalization, the datasets were subjected to quality control using NGS-QC (www.ngs-qc.org) to assess any influence of datasets quality in normalization. To avoid bias, clonal reads were excluded from the analysis in all datasets. The detailed materials and methods about the tools and their parameters used in different analysis followed are explained in the Additional file 1.

### Biological replicates

Epimetheus performance has been tested by using biological replicates of H3K4me3 mark datasets from nine different cell lines (GEO file GSE26320) [18]. The comparison of biological replicates is a standard procedure to reveal the effect of normalization, as the datasets are expected to be highly similar but they may differ, for example, in enrichment amplitudes and signal-noise ratio. Indeed, some of these replicates exhibited significant differences in signal-noise ratios and for the number of enrichment sites. To illustrate the effect of linear and quantile normalization for profiles with variable signal-noise ratio, examples of an enriched region in GM12878 cell replicates and of average RCI plots around transcription start sites (TSSs) for two HMEC replicates with different levels of global enrichment (background, less, medium and high) are shown in the Additional file 1: Figures S2A and S2B (top panels), respectively. The biological replicates (Rep1 and Rep2) of GM12878 exhibited varying signal-noise ratios, whereas those of HMEC exhibited a similar background and similar levels of weakly enriched sites but revealed major differences for highly enriched sites. In such a situation, linear normalization fails to properly correct (see the relative sizes of left peaks in the two replicates; Additional file 1: Figure S2A and S2B; middle panel). Using quantile normalization Epimetheus adjusted such amplitude differences along with the signal-to-noise ratio disparity among samples given its ranking-based approach (Additional file 1: Figure S2A and S2B; bottom panel). This is most convincingly seen in the average RCI TSS plots displayed in the Additional file 1: Figure S2B. While no major differences are seen for bins with low read counts, the TSS plots before and after normalization reveal major amplitude differences at medium (maximum RCIs between 25 and 50) and high (maximum RCIs above 50). An illustrative MA transformation plot displays the overall transition effect of normalization between the replicates with perfect LOESS line fit (Additional file 1: Figure S2C).

**Fig. 1:**
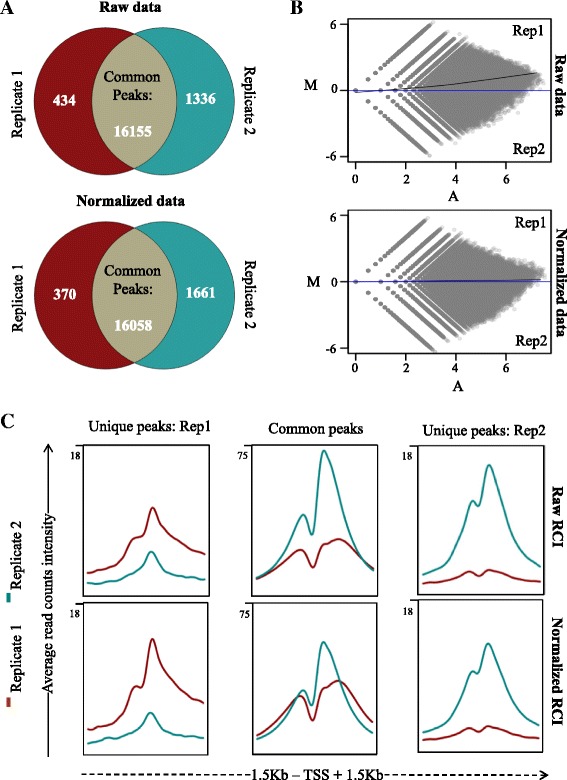
Effects of data normalization. **a** Pie charts illustrating the changes in number of common and replicate-specific promoter-associated H3K4me3 peaks for HepG2 cell line datasets (GSM646364; GSM646365) before and after normalization (*Blue*: Rep1-specific peaks, *Red*: Rep2-specific peaks and *Green*: Peaks common between replicates). While mostly peaks overlap rate are conserved, some changes are observed post normalization in less enriched peaks, thus influencing peak calling thresholds. **b** An illustrative MA transformation plot shows the overall transition of RCI differences between replicates before and after normalization. The LOESS fit line (*blue*) shows the overall correction change after normalization. **c** Average RCI plots over annotated promoters (TSS with flanking regions of 1.5Kb) show that significant amplitude difference exists with peaks that are common between replicates (*Blue*: Rep1 and *Red*: Rep2). However, after normalization such amplitude differences are corrected and replicate-specific enrichments become more distinctive

To evaluate the consequence of quantile normalization on a regular ChIP-seq peak calling approach, MACS peak calling was performed for the H3K4me3 HepG2 dataset with raw and normalized BED files (GSM646364; GSM646365). While normalization led to very small differences in the number of identified peaks (Fig. 1a), which was generally seen for less enriched sites, the correction of the overall amplitude, particularly towards higher read counts, was very obvious from the LOWESS fit line in the MA transformation plots of the raw and normalized data between replicates (Fig. 1b). Notably, in TSS plots the few replicate 1-specific peaks were enhanced by normalization both in number and intensity, and exhibited also weak (but apparently not peak caller-identified) signals in replicate 2, while the amplitude of unique peaks in replicate 2, which were invisible in both the raw and normalized replicate 1 TSS plots, got decreased (Fig. 1c). In contrast, the common peaks were in both replicates adjusted in opposite directions.

We conclude from these studies with biological replicates, which should in principle generate identical profiles that normalization results in a significant adjustment of peak intensities and increased the accuracy of identifying *bona fide* common peaks.

### Chromatin state analysis and Peak calling with normalization

**Fig. 2:**
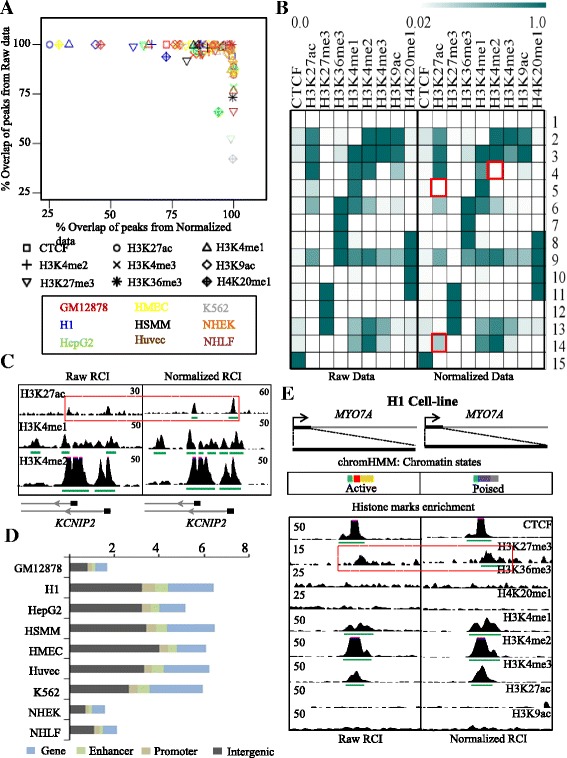
Chromatin state analysis using ChromHMM. **a** Illustration of peak consistency between raw and normalized data for nine histone marks that were used for chromatin state analysis for nine different cell lines, as indicated. X and Y-axis of the plot are the percentage of peaks overlapping between normalized and raw data, respectively. The least overlapping rate was observed for the H3K27ac profile of H1 cells, where all the peaks from raw data (100%) were retained post normalization but only 25% of peaks from normalized data overlapped with raw data peaks showing that additional peaks were identified post normalization. As for the H3K27ac profile of H1 cells, the poor overlap between peaks predicted from raw and normalized profiles was generally due to either poor quality and/or low coverage. **b** Emission parameters of ChromHMM describing chromatin state differences between raw and normalized peaks. Though the predicted chromatin states were conserved, three significant differences in enrichment levels are highlighted as *red-framed* boxes. **c** An example region illustrating the change after normalization of chromatin state 14 in Fig. 2b, where H3K27ac peaks become prominent after normalization. **d** Stacked bar chart indicating the percentage of chromatin state annotations per bin that changed upon normalization. While the GM12878, NHEK and NHLF datasets show few changes after normalization, the other datasets show more than 5% changed bin annotations. **e** Illustration of change in chromatin state annotation for the *MYO7A* locus using the same dataset processed with ChromHMM; note that the *MYO7A* promoter was annotated ‘active’ from the raw data and changed to ‘poised’ post normalization, which correlates perfectly with the absence of gene expression [Encode data: ENCSR962TBJ]

To illustrate Epimetheus’ performance in multi-profile integrative analysis, chromatin state analysis was performed using ChromHMM [19] with published data sets for nine histone marks of nine cell-lines (GSE26320) before and after normalization. Biological replicates of each sample were merged into one to increase the coverage. To consider also local background variation, which is ignored in the Poisson distribution-based ChromHMM, peak calling was carried out on raw and normalized data and identified peak regions were used for chromatin state analysis. A comparison of the peaks identified in raw and normalized data sets revealed that seven profiles showed at most 50% peak overlap, while the other profiles had a higher overlap rate (Fig. 2a). Interestingly, the datasets that show less overlaps between raw and normalized data were either of poor data quality (as assessed with NGS-QC Generator) or had a low coverage. For example, the H3K27ac profile of H1 cells for which only 25% of normalized data peaks overlapped with raw data peaks was constructed from only 17 M reads and its quality was rated CCD by NGS-QC (AAA is highest and DDD lowest quality). Similarly, several other datasets that showed a poor rate of overlaps were of either poor quality or coverage, highlighting the importance of quality control prior to analysis.

In general, the raw and Epimetheus-normalized data revealed the same overall prediction of chromatin states (Fig. 2b and c). However, in depth comparison showed that 2–7% of the genomic bins changed chromatin annotation post normalization; a handful of these were located in regulatory or gene regions (Fig. 2d). While the GM12878, NHEK and NHLF cell lines exhibited very few changes, the other cell-lines showed more than 5% of chromatin annotation changes. Importantly, some of these corrected a false-positive state annotation observed in raw data. For example, the chromatin state annotation at the *MYO7A* gene in H1 cells changed from “active” to “poised”; this is due to a more prominent enrichment of H3K27me3 after normalization (Fig. 2e). Importantly, transcriptome data from ENCODE [20] confirm that no *MYO7A* is expressed in H1 cells, which correlates well with the chromatin state annotation assessed after, but not with the one before normalization.

Together the above data reveal that normalization in general does not alter chromatin state annotation by ChromHMM but about 5% of them can be affected, thus leading to changes in the prediction of the functional states of a particular gene. In such cases normalization by Epimetheus improves chromatin state predictions.

### Temporal epigenetics dynamics during retinoic acid-induced F9 cell differentiation

**Fig. 3:**
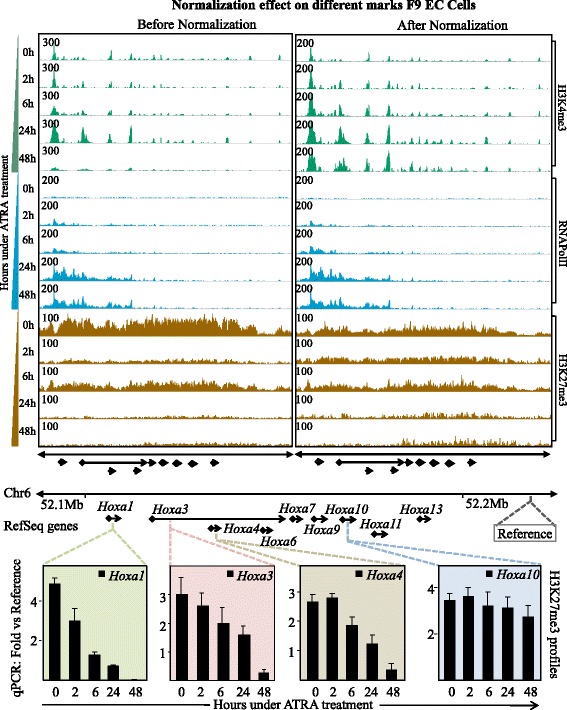
Signal intensity profiles of H3K4me3, H3K27me3 and RNAPolII enrichments at the *Hoxa* cluster; shown is the temporal signal evolution from consecutive ChIP-seq experiments during retinoic acid-induced differentiation of F9 cells. Most of the genes in *Hoxa* cluster have been shown to follow collinear gene activation pattern during differentiation with gradual increase of active marks and decrease of repressive marks. However, such pattern was not apparent from the raw RCI profiles. Data normalization resulted in the expected gradual spatio-temporal decrease of the H3K27me3 profile and concomitant increase of H3K4me3 & RNA PolII intensity profiles. The bottom panels reveal ChIP-qPCR analyses for H3K27me3, thus validating the normalization. Specifically *Hoxa1*, *Hoxa3* and *Hoxa4* genes - but not the *Hoxa10* gene –follows a collinear gene activation pattern, as observed in both the normalized data and the qPCR results

We then evaluated Epimetheus performance for time-series data, where a distinct gradual gain or loss of the signal amplitudes is expected. For this we used the well-characterized F9 mouse embryonal carcinoma (EC) cell model, which differentiates upon retinoic acid treatment [21]. Cells were grown as described [22, 23] and collected after 0 h, 2 h, 6 h, 24 or 48 h treatment with all-trans retinoic acid (RA). Each of the collected samples was used for assessing the epigenetic status by profiling the repressive histone modification mark H3K27me3, the transcriptionally active modification mark H3K4me3 and recruitment of RNA polymerase II [22]. It has been reported that the *Hoxa* cluster exhibits a collinear gene activation pattern during differentiation with a gradual gain of H3K4me3 and PolII recruitment, concomitant with a loss of H3K27me3 over the time [24, 25]. However, the statistical analysis of the raw data displays a rather non-uniform disparity for enrichment among samples and the profiles of the *Hoxa* cluster for the three targets did not show a clear temporal development over time points when raw data were used (particularly apparent for the 0 h, 2 h and 6 h time points in Fig. 3, top left panel). However, after normalization by Epimetheus, the H3K27me3 mark displayed a gradual decrease, whereas the active H3K4me3 mark and PolII recruitment showed a gradual temporal gain in signal intensities (right panel), both of which is entirely consistent with the previously described collinear gene activation pattern.

To further support the normalization results, we validated the H3K27me3 enrichment levels on various regions of the *Hoxa* cluster using quantitative PCR (qPCR). As illustrated in Fig. 3 (bottom panels), the qPCR results fully support the results obtained after normalization by Epimetheus. We conclude that normalization of temporal epigenetic data is imperative for unbiased data analysis.

## Discussion

Here we have demonstrated the effect of quantile normalization on a variety of datasets and show that normalization is imperative when performing a comparative analysis of the relative signal amplitude levels of NGS-profiles. Even though normalization appears to have a minor effect on the global data analysis by peak-finding algorithms, we show here that (i) normalization minimizes amplitude differences in replicates and increases confidence in the temporal evolution of signal amplitude of enrichment patterns and (ii) improves chromatin state annotations when using binarized enrichment data, as with ChromHMM.

In contrast to existing normalization tools, Epimetheus provides analytical outputs compatible with a variety of downstream analyses and illustrative plots. Indeed, previously described tools depend on peak caller predictions and/or external control datasets, the latter of which is generally the profile of the “input”, i.e. the whole cell extract (WCE) used for the ChIP. Given the diversity in available peak callers – each of which can be used with different parameters - and the potential bias introduced by a poor quality WCE, this approach could possibly lead to artefactual normalization. Specifically, even though control datasets exhibit in general an “ideal” (i.e., non-enriched) pattern, a few WCE controls show an artefactual enrichment-like pattern (examples are GSM788366 and GSM768313). Such patterns will significantly influence the normalization outcomes produced by tools like ChIP-norm.

In this context we have compared genome-wide and target-specific normalization using Epimetheus for genome-wide and a ChIP-norm (7)-like approach for normalization using a pre-selected bins displaying significant enrichment over the input control. We compared both the approaches by using datasets with different enrichment patterns (ChIP-seq datasets for the temporal evolution of H3K4me3, H3K27me3 and FAIRE-seq during retinoic acid-induced F9 cell differentiation; for details and dataset IDs, see Additional file 1: Supplementary Note). This comparison included an evaluation of the effect of the enrichment-factor of the selected population on normalization. Specifically, we asked, if ‘fold changes’ greater than 0, 1, 2, 3 or 4 between the IP and the input would influence the normalization. Indeed, this analysis revealed significant population-related biases relative to the genome-wide normalization of Epimetheus (Additional file 1: Figure S3); not only the normalization values are changed upon population selection (Additional file 1: Figure S3A) but also the resulting changes in signal intensities between samples from a temporal analysis (T48 vs. T0), which could lead to false interpretation of the temporal epigenome/chromatin changes during cell differentiation (Additional file 1: Figure S3B). Thus, while poor quality input samples can bias normalization approaches that use procedures similar to those of ChIP-norm, the genome wide normalization used by Epimetheus exhibited a robust performance across the datasets, as is revealed by MA plots, which display a perfect LOESS fit line (Additional file 1: Figure S3C, right panel). It is however apparent from this display that also the ChIP-norm-like procedure results in a major improvement of the datasets compared to the raw data (middle and left panels).

## Conclusions

To compensate for systematic technical variations between assays, normalization is requisite for any comparative multi-profile analysis of epigenome data provided that the datasets are indeed comparable with respect to their nature and quality (see above). In fact, datasets with systemic modifications of enrichment patterns or major differences in quality should obviously not be normalized. While most of the existing tools focus on normalization only for differential analysis, the present study with biological replicates and chromatin state analyses supports the necessity of normalization for any comparative, integrative or differential analysis. Relative to existing tools, the more robust and sophisticated options in Epimetheus are that (i) it can be customised to variety of requirements, (ii) it can be applied genome-wide or to specific regions (when justified), and (iii) it can exclude specific regions, such as repetitive elements, which would bias global normalization. We and others have shown previously that linear scaling-based tools cannot correct for the technical variations in ChIP-seq data. Other less user-friendly non-linear normalization tools are restricted to specific regions and their outputs cannot be usable easily for downstream analyses, such as binarized chromatin state annotations. For all these reasons, we have used Epimetheus in previous studies [22, 26, 27] and recommend Epimetheus for non-linear normalization with scalability for various downstream analysis pipelines.

## Additional file

Additional file 1: Figure S1. Scheme of the workflow of Epimetheus with illustrative plots. **Figure S2:** Epimetheus-based normalization of biological replicates using GSE26320. **Figure S3:** Comparison of normalization using genome-wide (Epimetheus) and ChIP-norm-based approaches. Supplementary note: Detailed summary of the Epimetheus methodology and the comparative studies, and of the datasets used. (PDF 723 kb)
