# Supplementary material for: Epimetheus - a multi-profile normalizer for epigenomic sequencing data
Source: BMC Bioinformatics. 2017 May 12;18:259. doi: 10.1186/s12859-017-1655-3 (PMC5429578; doi:10.1186/s12859-017-1655-3)
Supplement: Additional file 1: Figure S1. — Scheme of the workflow of Epimetheus with illustrative plots. Figure S2: Epimetheus-based normalization of biological replicates using GSE26320. Figure S3: Comparison of normalization using genome-wide (Epimetheus) and ChIP-norm-based approaches. Supplementary note: Detailed summary of the Epimetheus methodology and the comparative studies, and of the datasets used. (PDF 723 kb) [file 12859_2017_1655_MOESM1_ESM.pdf]

# Additional file 1

## Epimetheus - A multi-profile normalizer for epigenomic sequencing data

Mohamed-Ashick M. Saleem, Marco-Antonio Mendoza-Parra, Pierre-Etienne Cholley, Matthias Blum and Hinrich Gronemeyer

Equipe Labellisée Ligue Contre le Cancer, Department of Functional Genomics and Cancer, Institut de Génétique et de Biologie Moléculaire et Cellulaire, Centre National de la Recherche Scientifique UMR 7104, Institut National de la Santé et de la Recherche Médicale, U964, Université de Strasbourg, Illkirch, France

| | |
|---|---|
| **Additional file 1: Figure S1** | Scheme of the workflow of Epimetheus with illustrative plots |
| **Additional file 1: Figure S2** | Epimetheus-based normalization of biological replicates using GSE26320 |
| **Additional file 1: Figure S3** | Comparison of normalization using genome-wide (Epimetheus) and ChIP-norm-based approaches to monitor F9 cell differentiation using epigenome and PolII readouts from consecutive ChIP-seq assays |
| **Supplementary Note** | Detailed summary of the Epimetheus methodology and the comparative studies, and of the datasets used |

# Supplementary Figures

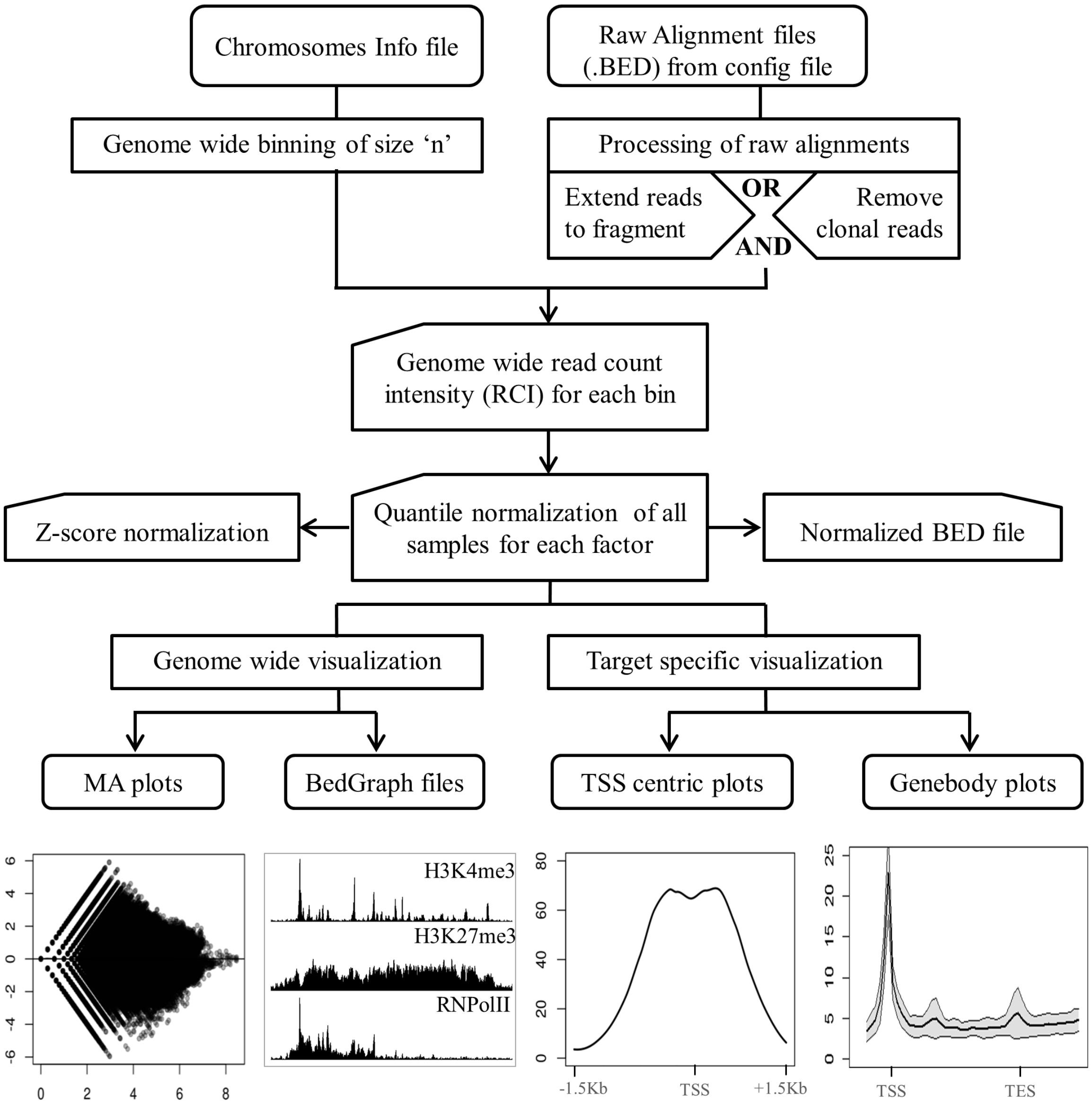


**Supplementary Figure 1.** A scheme of the workflow of Epimetheus with illustrative plots.

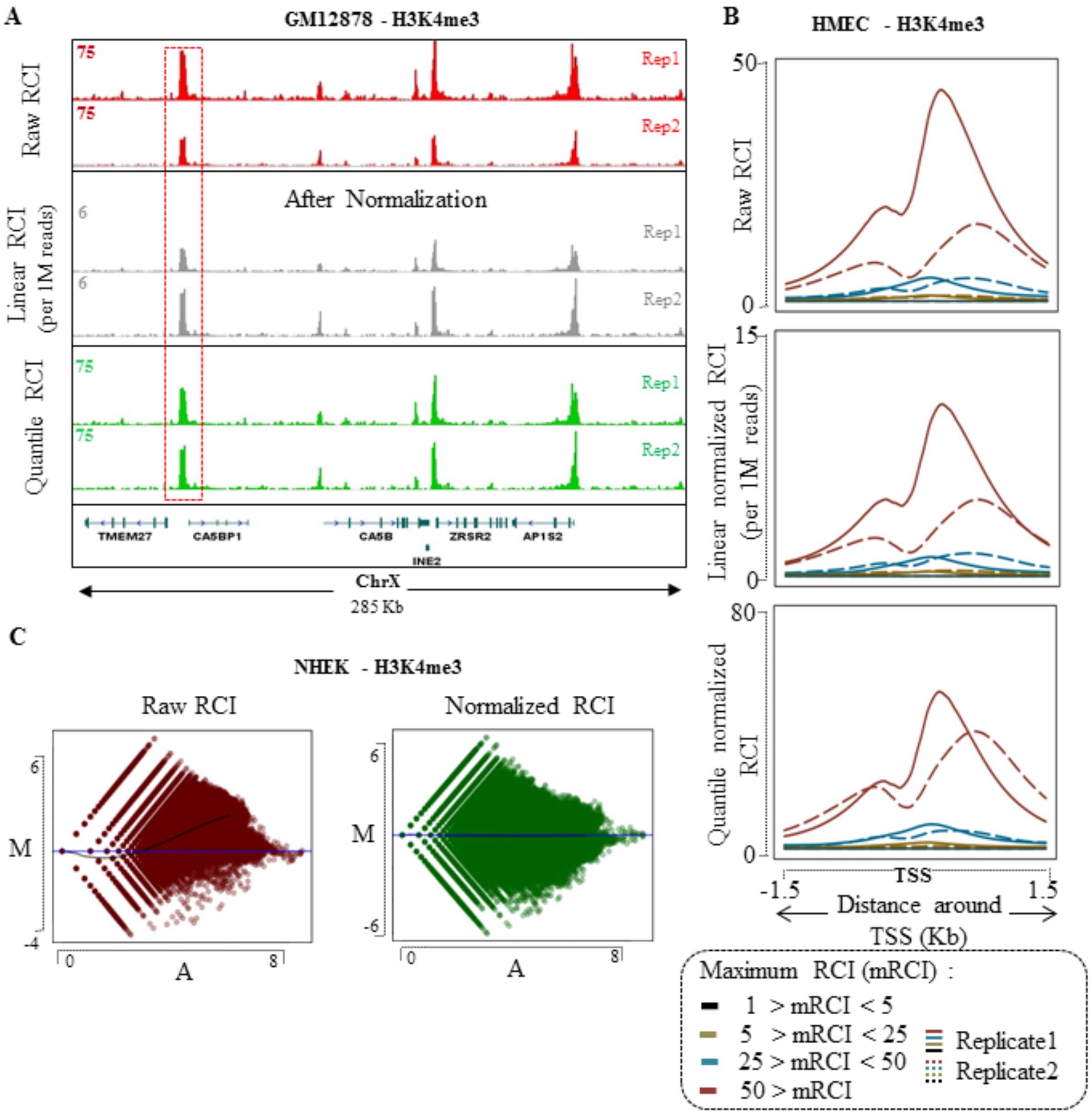


**Supplementary Figure 2.** Epimetheus normalization of biological replicates using GSE26320. (**A**) Signal intensity display of H3K4me3 profiles illustrating the effect of quantile normalization compared to linear normalization between replicates exhibiting different background-to-signal enrichment levels. In raw RCI (red), Rep1 displays higher amplitude than Rep2 which is corrected to similar scale after quantile normalization (green). However, due to varying signal-noise ratio, usage of linear normalization resulted in artifactual correction, where Rep2 displays higher amplitude level than Rep1 (grey). (**B**) Comparison of TSS centric plots (with flanking 1.5Kb) of replicates before (top panel) and after normalization stratified into four intensity levels; the corresponding colour code is depicted below the plot. While background and less enriched regions are very similar in the replicates, a major disparity was observed between the replicates for the highly enriched regions. Linear correction (middle panel) failed to correct such differences and resulted in a similar pattern, whereas quantile normalization (bottom panel) corrected the differences among highly enriched regions without affecting the less enriched/background regions. (**C**) MA transformation plot between replicates of NHEK cell-line displaying a global convergence of replicates datasets upon normalization. The perfect LOESS line fit (black) reveals correction of the technical variation between replicates (Red: Raw data; Green: Normalized data).

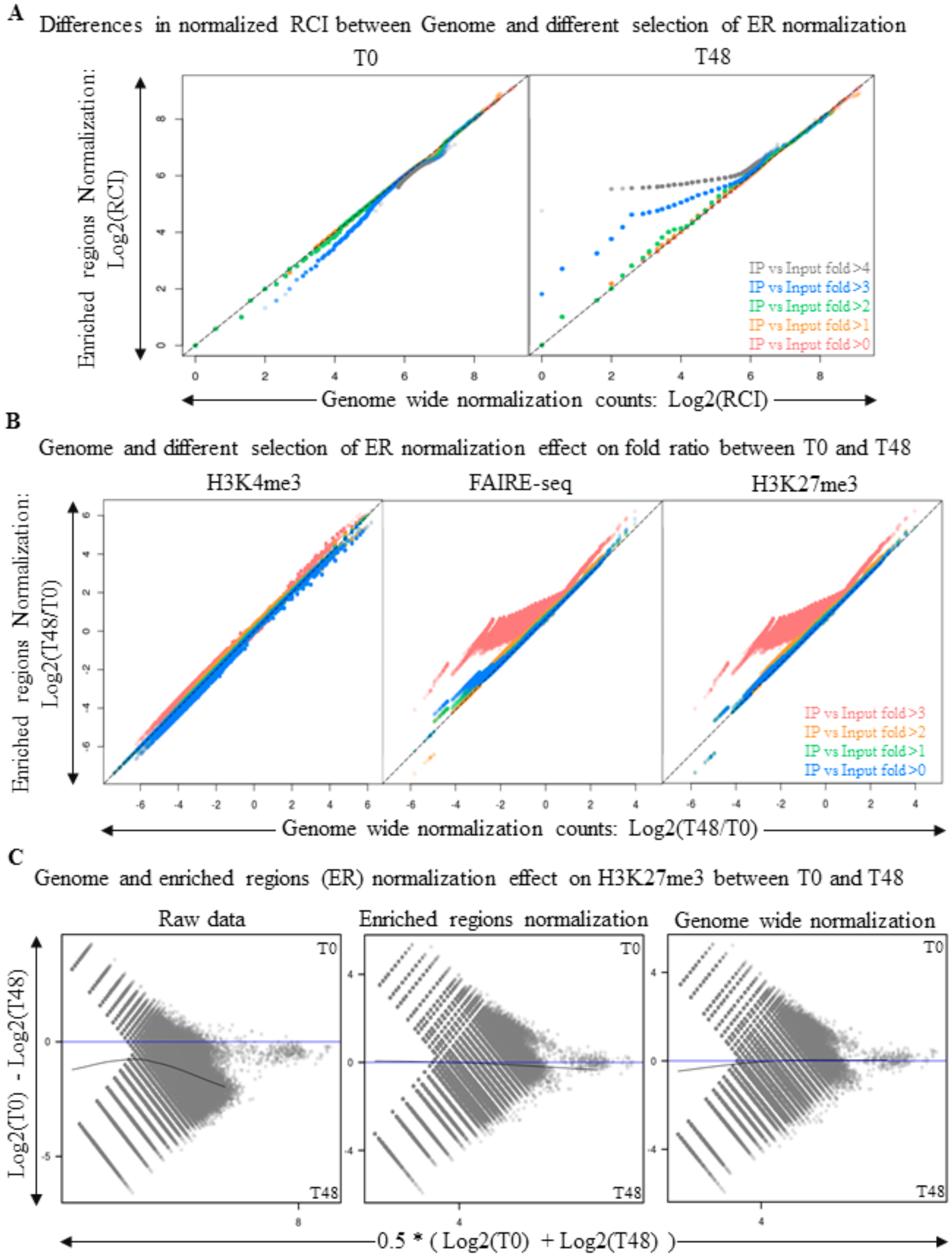


**Supplementary Figure 3.** Comparison of normalization using genome-wide (Epimetheus) and ChIP-norm-based approaches to monitor F9 cell differentiation using epigenome and PolII readouts from consecutive ChIP-seq assays (for a detailed description, see Supplementary Note). (**A**) Illustration of changes obtained by normalization with the Epimetheus *vs.* ChIP-norm approaches. Comparison of genome-wide (X-axis, where RCI are represented in log2) with enriched regions ('ER')-based normalized values (Y-axis, where RCI are represented in log2) using different ratios of IP *vs.* Input for the selection of the 'ER populations' in the ChIP-norm approach. Note the major divergence of the correlation plot from the diagonal when stringent criteria (fold change >3) were applied in the ChIP-norm approach to select 'ER populations'. (**B**) Effect of the selection stringency of 'ER populations' in differential analysis. Plot of the retinoic acid-induced changes between T0 and T48 datasets for H3K4me3, FAIRE-seq and H3K27me3; the Y-axis plots the log ratio of T48/T0 read count intensities obtained after normalization at different 'IP *vs.* Input' ratios to identify enriched regions according to the ChIP-norm approach relative to the same regions obtained after genome-wide Epimetheus-based normalization (X-axis). (**C**) MA plots revealing the normalization effect when using ChIP-norm-based (IP vs input fold >1) and Epimetheus-based normalization procedures. A LOWESS fit line (black) is included in each plot.

# Supplementary Notes

### Datasets

Datasets for the comparative and validation analysis on nine cell lines (GSE26320 [1]) were downloaded from GEO; F9 cell data have been deposited at GEO (GSE68291).

### Processing and alignment of NGS data

For chromatin state analysis on nine cell lines, aligned BED files were directly downloaded and used for the analysis. For F9 cell data, reads were aligned against mm9 genome using Bowtie (v 1.1.1) [2]. Clonal reads were removed before analysis and reads were elongated to 200bp for both analyses.

### Peak Calling

For peak calling, MACS [3] was used with 1e-9 p-value and no-model option. SICER [4] was used to identify broad histone marks islands (H3K27me3, H3K36me3 and H4K20me1) in the nine cell line chromatin state analysis.

### ChromHMM

We performed ChromHMM with enriched regions identified using peak calling as input. Peaks from both raw and normalized BED files were provided as input separately and ChromHMM was performed with 400 iterations to predict 15 states using custom scripts for annotation.

### Comparison of Epimetheus (genome-wide) with ChIP-norm (enriched regions-based) normalization approaches

To illustrate the superiority of genome-wide normalization over target-specific normalization, we compared both methods using Epimetheus for genome-wide and a ChIP-norm [5]-like approach for target specific approaches. We compared both the approaches on datasets with different enrichment patterns like H3K4me3, H3K27me3 and FAIRE-seq.

As ChIP-norm is written in MATLAB (commercial software), we could not use it directly for comparison; instead, we wrote scripts following the outline of ChIP-norm workflow. F9 cell datasets were used to compare T0 (non-differentiated cells and time point 0) and T48 (48h of induction with all-*trans* retinoic acid to induce differentiation, as described by Mendoza-Parra et al [6]) samples are used from three different marks H3K4me3, H3K27me3 and FAIRE-seq. The same “input” profile was used for both the time points on ChIP-norm-like normalization approach. Three main steps in this approach is 1) exclusion of background regions 2) Input and IP are normalized together using quantile and 3) identifying enriched regions based on fold change of Input vs IP. First steps are similar as in Epimetheus and Xu *et al*., where genome is binned into small windows and read counts intensity (RCI) matrix is built for each sample. We then used Poisson distribution to identify number of reads that can be randomly filled in bins by using total number of reads, effective genome size (with P-value of 0.995). It is followed by applying quantile normalization between input and IP to bring them to same scale to perform fold change analysis to identify enriched bins. In general, fold change >1 is used to identify ER whereas we altered this criterion to different ranges to see the influence of

population selection in quantile normalization. We selected ‘fold changes’ greater than 0, 1, 2, 3 or 4 (Supplementary Figure 3A). Fold change 0 would include bins in IP that have even one read count more than the corresponding input bin, while the other ‘fold changes’ consider enrichment based on ratios. To compare ‘genome-wide’ and ‘ER only’ normalization, we considered for each ‘fold change’ only bins from ‘genome-wide normalization’ that corresponded to the ‘ER only’ bins. Also, to compare the effect of population selection on differential analysis result between samples, we selected ‘input vs IP fold change >3’ bins as reference, as ‘fold change >4’ has very few bins. We noted that increasing the ‘fold change’ of ‘input vs IP’ for identifying enriched regions increased also the discrepancy in differential enrichment (fold change) between samples (Supplementary Figure 3B). Though the more discrepancy is observed on very stringent fold change criteria, it is also evident that it depends on enrichment pattern and its population similarity/diversity between samples. As it is illustrated in Supplementary Figure 3B, sharp enrichment H3K4me3 is relatively less affected than FAIRE-seq data or ‘broad enrichment’, like that seen for H3K27me3.

**Plots**

All the plots were generated using custom R scripts; ChromHMM chromatin states heat-map was generated using MeV (Multiple Experiment Viewer) suite and intensity profiles display was generated using UCSC genome browser [7] and IGV [8].

**qPCR analysis for F9 data**

Details of oligonucleotides used for the qPCR validation of the data on *Hoxa* cluster region confirming the normalized results.

| **Oligo Name** | **Sequence 5' to 3'** | **Scale (µmole)** | **Purification** |
|---|---|---|---|
| HoxA_Rctrl_F | GCTGCAGGGGATAAACACAT | 0.05 | DST |
| HoxA_Rctrl_R | GCTGGAACATTAAGGCCAAA | 0.05 | DST |
| HoxA10_F | ATGAGCGAGTCGACCAAAAA | 0.05 | DST |
| HoxA10_R | ATGTCAGCCAGAAAGGGCTA | 0.05 | DST |
| HoxaA4_F | TCCTCGAAAGGAGGGAACTT | 0.05 | DST |
| HoxaA4_R | CGACACCGCGAGAAAAATTA | 0.05 | DST |
| HoxA3_F | GTCTGGAGTTGGGGGATTTT | 0.05 | DST |
| HoxA3_R | ACCTAGCCTCCAGACCCTGT | 0.05 | DST |

**Supplementary References:**


1. Ernst J, Kheradpour P, Mikkelsen TS, Shoresh N, Ward LD, Epstein CB*, et al.* Mapping and analysis of chromatin state dynamics in nine human cell types. Nature. 2011; 473:43-49.

2. Langmead B, Trapnell C, Pop M, Salzberg SL. Ultrafast and memory-efficient alignment of short DNA sequences to the human genome. Genome Biol. 2009; 10:R25.

3. Zhang Y, Liu T, Meyer CA, Eeckhoute J, Johnson DS, Bernstein BE*, et al.* Model-based analysis of ChIP-Seq (MACS). Genome Biol. 2008; 9:R137.

4. Zang C, Schones DE, Zeng C, Cui K, Zhao K, Peng W. A clustering approach for identification of enriched domains from histone modification ChIP-Seq data. Bioinformatics. 2009; 25:1952-1958.

5. Nair NU, Sahu AD, Bucher P, Moret BM. ChIPnorm: a statistical method for normalizing and identifying differential regions in histone modification ChIP-seq libraries. PLoS One. 2012; 7:e39573.

6. Mendoza-Parra MA, Walia M, Sankar M, Gronemeyer H. Dissecting the retinoid-induced differentiation of F9 embryonal stem cells by integrative genomics. Mol Syst Biol. 2011; 7:538.

7. Kent WJ, Sugnet CW, Furey TS, Roskin KM, Pringle TH, Zahler AM*, et al.* The human genome browser at UCSC. Genome Res. 2002; 12:996-1006.

8. Thorvaldsdottir H, Robinson JT, Mesirov JP. Integrative Genomics Viewer (IGV): high-performance genomics data visualization and exploration. Brief Bioinform. 2013; 14:178-192.
